# Trait‐based adaptability of *Phragmites australis* to the effects of soil water and salinity in the Yellow River Delta

**DOI:** 10.1002/ece3.7925

**Published:** 2021-07-28

**Authors:** Dayou Zhou, Yuehan Ni, Xiaona Yu, Kuixuan Lin, Ning Du, Lele Liu, Xiao Guo, Weihua Guo

**Affiliations:** ^1^ Shandong Provincial Engineering and Technology Research Center for Vegetation Ecology School of Life Sciences Institute of Ecology and Biodiversity Shandong University Qingdao China; ^2^ State Environmental Protection Key Laboratory of Estuarine and Coastal Environment Chinese Research Academy of Environment Sciences Beijing China; ^3^ College of Landscape Architecture and Forestry Qingdao Agricultural University Qingdao China

**Keywords:** electrical conductivity, functional traits, life strategies, plasticity, soil water content

## Abstract

*Phragmites australis* is the dominant species in the Yellow River Delta and plays an important role in wetland ecosystems. In order to evaluate the relationship between phenotypic variation and environmental factors, explore how functional traits respond to changes in electrical conductivity and soil water content, and reveal the ecological strategies of *P. australis*, we investigated the ecological responses of *P. australis* to soil properties based on 96 plots along the coastal–inland regions in the Yellow River Delta of China. Within the range of soil water content (SWC, 9.39%–36.92%) and electrical conductivity (EC, 0.14–13.29 ms/cm), the results showed that (a) the effects of salinity were more important than the soil water content for the characterization of the morphological traits and that plant functional traits including leaf traits and stem traits responded more strongly to soil salinity than soil water content; (b) compared with morphological traits such as average height and internode number, physiological traits such as SPAD value, as well as morphological traits closely related to physiological traits such as specific leaf area and leaf thickness, showed stronger stability in response to soil water and salinity; and (c) under the condition of high electrical conductivity, *P. australis* improved its water acquisition ability by increasing indicators such as leaf water content and leaf thickness. In addition, with the increase in plant tolerance to stress, more resources were used to resist external stress, and the survival strategy was inclined toward the stress tolerator (S) strategy. Under low EC conditions, *P. australis* increased specific leaf area and leaf area for its growth in order to obtain resources rapidly, while its survival strategy gradually moved toward the competitor (C) strategy.

## Graphical abstract

In the Yellow River Delta, soil salinity was the main factor that influenced the functional traits of *P. australis*; the physiological traits are more stable than morphological traits in response to environmental variables; and competition and stress tolerator were the main strategies of *P. australis*.

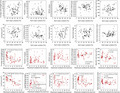

## 1 INTRODUCTION

Plant functional traits have been useful in answering many important ecological questions at a range of scales (Mason and de Bello, 2013; Pérez‐Harguindeguy et al., 2013), providing a tool for determining the feedback of plants under stressful conditions. Plant functional traits can be divided among groups into different types of traits that reflect similar responses to environmental factors or exert similar effects on community dynamic processes based on plant morphology and physiology (Duckworth et al., 2000; Guo et al., 2017). Morphological and physiological features can reflect plant ecological strategies, with a shift among competitive, stress‐tolerant, and ruderal strategies (Chai et al., 2016; Spasojevic et al., 2014). Owing to their adaptability and plasticity in response to environmental gradients, individual plants have highly variable traits. The variation in plant functional traits determines the feedback of plants in response to various environmental factors (Guan et al., 2017; Li et al., 2014). Functional traits can be used to quantify a wide range of natural and anthropogenic disturbances (Wang et al., 2015; Chai et al., 2016).

Numerous studies have reported the relationships between plants and salinity in various habitats (González‐Alcaraz et al., 2014; McCoy‐Sulentic et al., 2017; Wang et al., 2012). These studies have greatly contributed to our understanding of the soil–plant interactions that benefit wetland ecosystem restoration. Salinity is one of the major environmental factors limiting plant growth and productivity (Sdouga et al., 2019). Salinity also affects multiple trait strategy dimensions, causes consequences for ecosystem functions (De Battisti et al., 2020), and leads to environmental filtering that drives plant community assembly processes (Yi et al., 2020). Plant growth is also highly dependent on soil water content, especially in arid and semi‐arid regions. Many researchers have reported that community structure (Pérez‐Ramos et al., 2012), species composition (Li et al., 2008), and vegetation growth (Gong, Li, et al., 2014; Gong, Zhu, et al., 2014; Yu et al., 2014) can be affected by soil water content.

Plants adapt to heterogeneous habitats through plasticity in growth strategies and functional traits, as well as the optimal allocation and trade‐offs of various traits (Donovan et al., 2014; Guan et al., 2017; Mason and de Bello, 2013). Competitor, stress tolerator, ruderal (CSR) theory is a prominent strategy scheme advanced by Grime (1977) and reviewed by Grime and Pierce (2012), in which the three principal strategies represent viable trait combinations. They have also been used to investigate and interpret community processes, such as succession and the relationship between species richness and productivity (Caccianiga et al., 2006; Cerabolini et al., 2016). Although they are less precise than methods that consider whole‐plant traits, classification tools based on a few leaf traits have an advantage in that many measurements can be performed with minimal effort. Three core leaf functional traits, specific leaf area (SLA), leaf dry matter content (LDMC), and leaf area (LA), were used as criteria to determine the ecological strategies of the individual or population of the plant being studied (Pierce et al., 2013, 2017). This is a dimensionality reduction analysis based on the global TRY plant functional trait database (Kattge et al., 2011). Leaf area, a key determinant of the capacity to intercept light, is one of the most widely available indicators of the size spectrum (Díaz et al., 2016). Specific leaf area and leaf dry matter content are the most widely available traits, and they are highly representative of the opposite extremes of the economics spectrum (Pierce et al., 2017). The plant CSR strategy taxonomy goes beyond the previous research that focused on analyzing the plant ecological strategies of different species in communities (Pierce et al., 2017; Xu et al., 2019) or populations within species (May et al., 2017; Vasseur et al., 2018).

*Phragmites australis* has variable environmental adaptability and phenotypic plasticity (Yang et al., 2014), is widespread in both freshwater and brackish habits, is more likely to be found in sites where there is surface ground water discharge, and appears to access deeper, less saline water (Burdick et al., 2001; Guo et al., 2018; Veldhuis et al., 2019). *P. australis* is a salt‐tolerant plant (Mauchamp and Mésleard, 2001; Santos et al., 2016), and its broad range of adaptions to soil water content and salinity has led to the successful growth of *P. australis* in high‐salinity areas (Achenbach et al., 2013; Burdick et al., 2001; Guo et al., 2018). In order to explore the responses of functional traits of *P. australis* to soil water content and salinity, a field investigation was carried out in the Yellow River Delta. Here, we hypothesized that (a) the effects of soil water content and salinity were not equally important in characterizing the functional traits and (b) the ecological strategies of *P. australis* could be reflected by the classification of the intraspecific variation of functional traits and characterized under different forms of environmental stress.

## 2 MATERIALS AND METHODS

### 2.1 Study region and sampling sites

**FIGURE 1:**
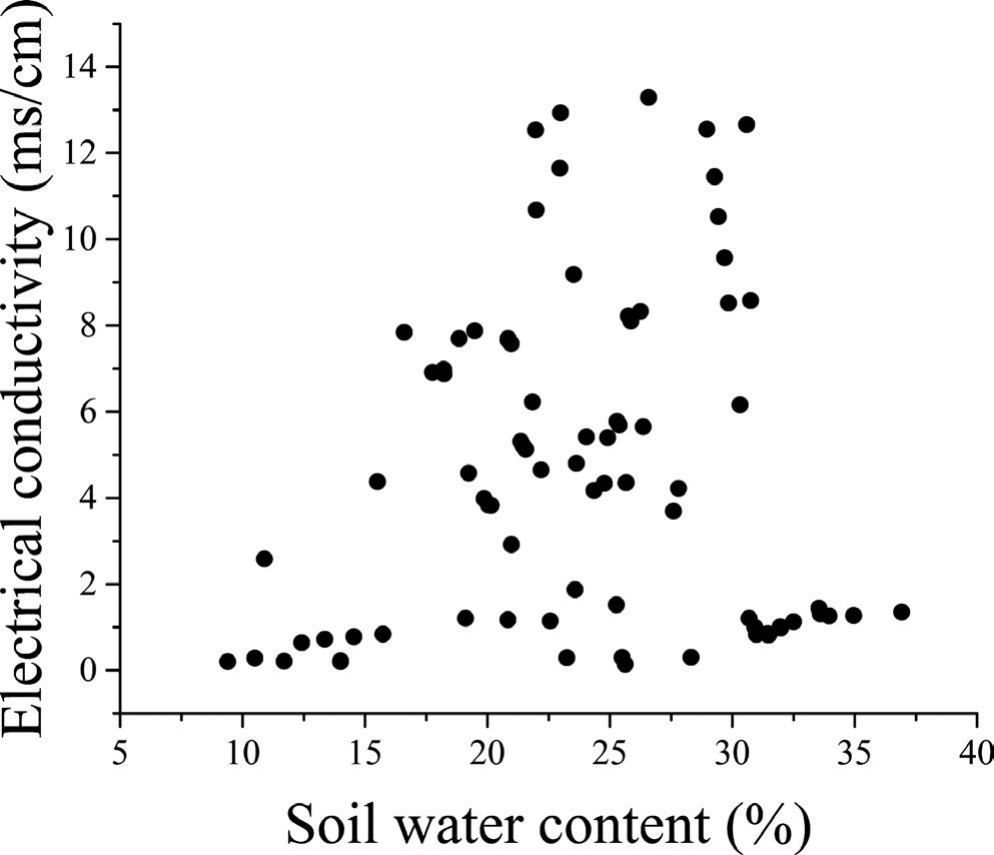
Distribution patterns of soil water content and electrical conductivity for 96 plots

The study site was located in the Yellow River Delta (36°55′–38°16′N, 117°31′–119°18′E) in Dongying, Shandong Province, northern China. The mean annual precipitation in the study area is approximately 628.6 mm, and the mean annual temperature is about 11.9°C (Zhou et al., 2020). The region is characterized by the strong temporal and spatial heterogeneity of soil water, and the salinity gradients are simultaneously influenced by river water, ground water, and seawater (Zhou et al., 2020). To describe and characterize the *P. australis* influenced by the patterns and processes under different gradients of soil water content and electrical conductivity, 96 plots (1 × 1 m) were selected randomly along the coastal–inland regions (37°43′–38°05′N, 118°41′–119°13′E) in the Yellow River Delta in 2013. Samples with significant differences in soil water content and salinity gradients were chosen in typical *P. australis* communities with little human disturbance (Figure 1).

### 2.2 Measurements of soil properties and functional traits

Soil samples were obtained at a depth of 0–20 cm to measure soil water content (SWC) and electrical conductivity (EC). SWC was measured by drying soil samples at 105℃ to constant weight. SWC was calculated as follows:

(1) SWC=(wet soil weight‐dry soil weight)×100%/dry soil weight.

Soil samples were dug from the 0–20 cm soil layer to measure electrical conductivity (EC). Ten grams of grated dried soil was placed in an Erlenmeyer flask and mixed with a water to a soil volume ratio of 2.5:1. A magnetic stirrer was used to dissolve the salt, followed by a resting period of about 40 min. A conductivity meter (DDS12A, LIDA, China) was used to measure the EC in the upper suspension.

For each sample, 10 functional traits were measured, including average height, number of plants, soil plant analysis development (SPAD) values, leaf water content (LWC), specific leaf area (SLA), leaf area (LA), leaf thickness, internode number, internode height, and stem basal diameter. SPAD values were determined using a chlorophyll meter (SPAD‐502PLUS, Konica Minolta, Japan). LA was analyzed using a broadleaf analysis system (WinFolia pro LM2400P, Regent) through scanning with a Canon scanner (Canon 2000).

SLA was calculated using the following equation:

(2) SLA=LA/LDM,

where LDM was the dry mass of leaves, which were oven‐dried at 80°C to constant weight and weighed (g).

LWC was calculated using the following equation:

(3) LWC=(LFM‐LDM)/LFM=1‐(LDM/LFM),

where LFM was the fresh mass of leaves (g).

Leaf dry matter content (LDMC,%) was calculated using the following equation:

(4) LDMC=1‐LWC.

Leaf thickness (mm) was measured by a micrometer caliper at the widest point, avoiding the midrib, and the stem basal diameter was determined using a vernier caliper.

The CSR model was calculated using the method developed by Pierce et al. (2017). The method is based on an algorithm that combines data for LA, SLA, and LDMC, and has been shown to reliably position the species in the CSR scheme. LA data were standardized using the maximum value, followed by square root transformation (Podani, 2007). LDMC data were logit‐transformed (Warton & Hui, 2011), and SLA data were log‐transformed (Pierce et al., 2017). We calculated CSR scores for each accession using the average trait value per experiment using the calculation table provided in the supplementary information (Appendix S1).

### 2.3 Data analysis

A one‐way analysis of variance was conducted to identify differences in environmental traits and functional traits. The normality was tested prior to one‐way analysis. The primary statistical analyses, functional traits relationship, partial correlation, and curve estimation were performed using SPSS 20.0 (SPSS Inc., 2011). The CSR model was calculated by the globally calibrated CSR analysis tool StrateFy (Pierce et al., 2017) (Appendix S1). Figures were plotted using Origin Pro 2016 (Originlab Co., 2016) and R (R Development Core Team, 2013).

## 3 RESULTS

### 3.1 Trade‐off among *P. australis* traits

**FIGURE 2:**
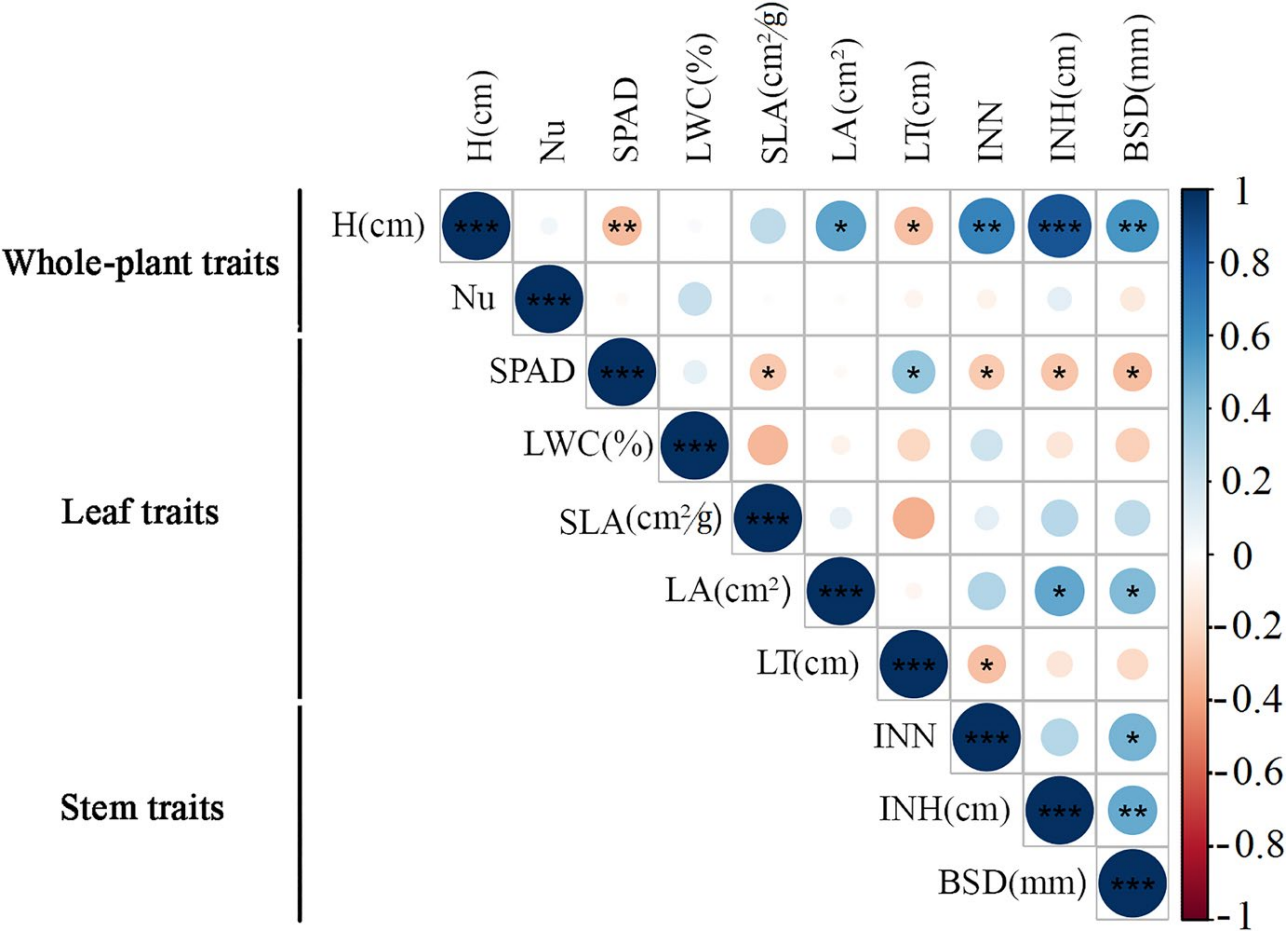
Trade‐off among *P. australis* traits

In order to investigate the trade‐off among *P. australis* traits, the Pearson correlation coefficients were calculated (Figure 2). The height of *P. australis* had obvious positive correlations with the leaf growth traits, such as SLA (*p* < .01) and leaf area (*p* < .05); and the stem traits included the internode number (*p* < .01), internode height (*p* < .001), and stem basal diameter (*p* < .01). Positive correlations were founded among these traits. The SPAD and leaf thickness had significant negative correlations with other leaf and stem traits, while SPAD and leaf thickness had a positive correlation (*p* < .05). The number of plants had no relationship with other traits (*p* > .05).

Pearson correlation coefficients are shown in the table. Asterisks indicate a significant correlation between environmental variables and functional traits (two‐tailed) (* *p* < .05; ** *p* < .01; and *** *p* < .001). Abbreviations: Whole‐plant traits (H, average height and Nu, number of plants); leaf traits (SPAD, SPAD values; LWC, leaf water content; SLA, specific leaf area; LA, leaf area; and LT, leaf thickness); and stem traits (INN, internode number; INH; internode height; and BSD, stem basal diameter).

### 3.2 Response of functional traits to environmental variables

Partial correlations were conducted to determine the relationships between environmental variables and functional traits (Table 1). The results indicated that functional traits could better respond to variables for EC rather than SWC. The average height, SLA, leaf area, internode number, internode height, and stem basal diameter were significantly negatively correlated with EC (*p* < .01), while significantly positive relationships were only discovered between SWC and leaf water content, and between EC and leaf water content and leaf thickness (*p* < .05).

**TABLE 1:** Partial correlation coefficient matrix between environmental variables and selected functional traits (whole‐plant traits, leaf traits, and stem traits) of *P. australis*

Index	SWC		EC	
	*r*	*p*	*r*	*p*
Whole‐plant traits				
Average height	.204	.060	**−.563** ***	.000
Number of plants	.019	.862	.014	.901
Leaf traits				
SPAD values	−.123	.259	.063	.564
Leaf water content	.238*	.027	.228*	.035
Specific leaf area	.088	.423	**−.442** ***	.000
Leaf area	−.068	.5329	**−.619** ***	.000
Leaf thickness	−.047	.668	.232*	.031
Stem traits				
Internode number	.142	.193	−.349**	.001
Internode height	.075	.494	**−.524** ***	.000
Stem basal diameter	.072	.510	−.290**	.007

Note

Asterisks indicate a significant correlation between environmental variables and functional traits (two‐tailed) (* *p* < .05; ** *p* < .01; and *** *p* < .001). Bold values indicate the significant correlation between functional traits and environmental factors.

Abbreviations: EC, electrical conductivity; SWC, soil water content.

**FIGURE 3:**
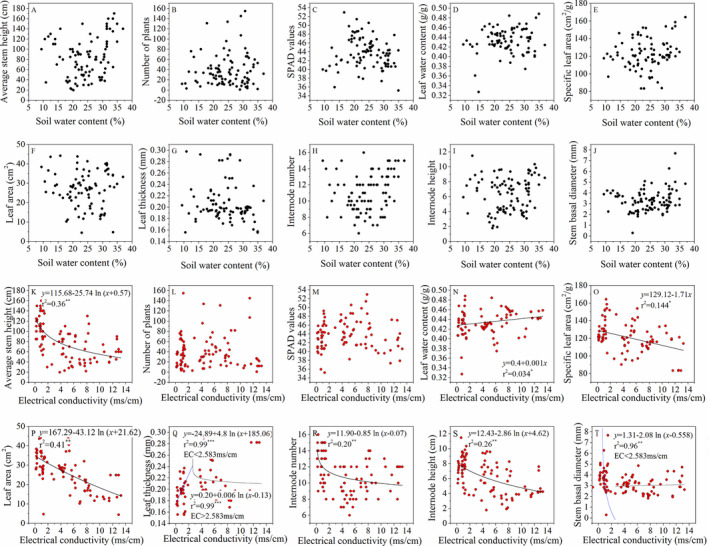
Regression relationship between functional traits of *P. australis* and soil water content. (a–j), electrical conductivity (k–t). a, k, average stem height; b, l, number of plants; c, m, SPAD values; d, n, leaf water content; e, o, specific leaf area; f, p, leaf area; g, q, leaf thickness; h, r, internode number; i, s, internode height; and j, t, stem basal diameter

Significant regression relationships were only observed between functional traits and EC, and these relationships were generally linear and logarithmic (Figure 3). For whole‐plant trait variables, significantly logarithmic relationships were only found between the EC and average height (*p* < .001) of *P. australis*. For leaf traits, significant correlations were discovered in the relationship between EC and leaf water content (*p* < .05), SLA (*p* < .01), leaf area (*p* < .001), and leaf thickness (*p* < .01). For stem traits, significant correlations were observed between EC and all selected stem traits, including internode number (*p* < .001), internode height (*p* < .001), and stem basal diameter (*p* < .01). Moreover, the relationships between EC and leaf thickness and stem basal diameter had an obvious inflection point (2.58 ms/cm). As the EC increased, the leaf thickness revealed a significant increase trend and then decreased (*p* < .01). With regard to the stem basal diameter, there were no significant relationships with EC when EC was higher than 2.58 ms/cm (*p* > .05).

### 3.3 Phenotypic plasticity in *P. australis*

Environmental factors and selected functional traits of *P. australis* varied greatly (Table 2), indicating the strong phenotypic plasticity and environmental tolerance of *P. australis*. The coefficient of variation of SWC was larger than 25%, while that of the EC was up to 87%. The amplitude of SWC (9.39%–36.92%) and EC (0.14–13.29 ms/cm) indicated the high tolerance of *P. australis* to environmental variables in the Yellow River Delta. The coefficients of variation of leaf traits were lower than 20%, except for the leaf area, while the whole‐plant traits and stem traits were higher than 20%.

**TABLE 2:** Statistical characteristics of environmental variables and functional traits for *P. australis*

Index	Minimum	Maximum	Mean	*SD*	CV (%)
Environmental variables					
Soil water content (%)	9.39	36.92	24.65	6.4	25.95
Electrical conductivity (ms/cm)	0.14	13.29	4.55	3.9	86.51
Whole‐plant traits					
Average height (cm)	20	170	82.73	37.67	45.54
Number of plants	1	352	42.36	44.91	106.01
Leaf traits					
SPAD values	22.2	55.53	43.45	4.46	10.25
Leaf water content (%) (g/g)	32.71	48.82	43.31	0.03	5.96
Specific leaf area (cm^2^/g)	83.46	164.41	121.48	16.88	13.89
Leaf area (cm^2^)	4.46	44.19	26.96	9.68	35.91
Leaf thickness (mm)	0.16	0.3	0.21	0.03	15.93
Stem traits					
Internode number	6	16	11.14	2.37	21.25
Internode height (cm)	1.77	11.5	6.34	2.26	35.59
Stem basal diameter (mm)	1.83	7.68	3.33	0.96	28.87

Abbreviations: CV, coefficient of variation; *SD*, standard deviation.

### 3.4 Ecological strategy of *P. australis* under different environmental factors

**FIGURE 4:**
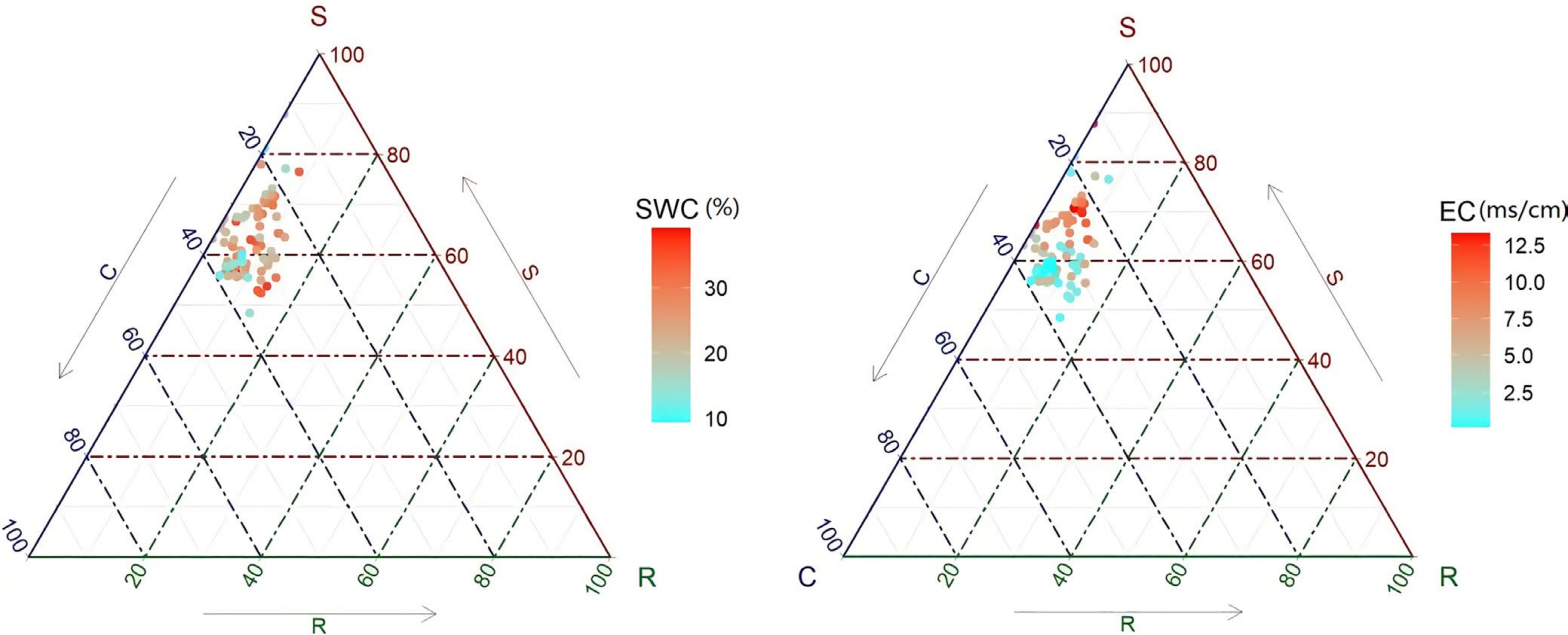
Ecological strategies of *P. australis* under different environmental conditions based on the 96 plots. SWC, soil water content; EC, electrical conductivity; C, competitor strategy; S, stress tolerator strategy; and R, ruderal strategy

Based on CSR scores, the ecological strategies of *P. australis* in the Yellow River Delta under different water and salinity conditions mainly comprised stress tolerator/competitor–stress tolerator (S/CS) (57.3%, C: S: R = 28.5:66.7:4.7), competitor–stress tolerator (CS) (27.1%, C: S: R = 36.0:57.3:6.7), stress tolerator/competitor–stress tolerator–ruderal (S/CSR) (11.5%, C: S: R = 29.9:57.8:12.3) strategies, and rarely competitor–stress tolerator/competitor–stress tolerator–ruderal (CS/CSR) (3.1%) and stress tolerator (S) (1%) strategies (Figure 4). The major strategies consisted of S/CS and CS strategies (84.4%). The three strategies of S/CS‐CS‐S/CSR formed an S‐C trade‐off strategy axis, indicating that the competition and stress tolerance strategies were dominant in the wetland ecosystems with high‐salinity conditions in the Yellow River Delta, while the R strategy was minor and mainly distributed in low‐salinity areas. However, with the decrease in soil EC and the increase in SWC, the C strategy of *P. australis* gradually increased, which enhanced the competition of *P. australis* with other plants in the communities.

## 4 DISCUSSION

### 4.1 Effects of environmental variables on functional traits of *P. australis*

Salinity stress resulted in obvious stunting in plants (Wang et al., 2016) and reduced leaf area expansion (Gong et al., 2020; Parida and Das, 2005). As previously reported, the height reduction of *P. australis* growth was a distinctive feature under salinity stress (Lissner and Schierup, 1997; Guo et al., 2018; Sdouga et al., 2019). In addition, many studies have reported that community structure (Pérez‐Ramos et al., 2012), species composition (Li et al., 2008), and vegetation growth (Gong, Li, et al., 2014) are affected by SWC. Generally, there is a nonlinear relationship with an obvious soil water content threshold value for most plants (Yu et al., 2012), and plant growth can be limited by both deficit and excess soil water.

Our results clearly verified the effects of salinity stress in *P. australis*. Only the leaf water content was interacted with SWC and EC (Table 1), and other traits, including the average height, SLA, leaf area, leaf thickness, internode number, internode height, and stem basal diameter, showed a significant response to EC. The average height of *P. australis* was negatively correlated with EC. Salinity also affects plant leaves through reduced specific leaf area and leaf area (Figure 3), but increased leaf water content and leaf thickness enhance the ability to resist environmental stress. Stem traits play a vital role in the entire plant life cycle, through supporting aboveground organization, retaining water and nutrients, and conducting water and elements (Liu et al., 2016). Based on our results, the stem traits had positive correlations among themselves (Table 1), which were generally negatively correlated with EC (Figure 3). Under low EC values, the plants had a relatively high growth rate, with stem elongation, an increased number of sections, and thickened stem basal diameter, while they exhibited decreased leaf thickness to support more leaves in order to gain more photosynthetic energy and compete for resources.

Leaf thickness plays a key role in determining the physical strength of leaves. Leaf thickness increases under stress and plays a role in sustaining the leaf water content (Wu et al., 2008). Interestingly, our results indicated that there was a turning point (≈2.58 ms/cm) for the relationship between EC and leaf thickness and between EC and stem basal diameter. Leaf thickness demonstrated an increasing trend before 2.58 ms/cm, which then became negatively correlated with EC beyond the turning point. In contrast, the stem basal diameter revealed a negative correlation before the turning point and then remained relatively stable. Salt stress increased the leaf thickness in order to adapt to increasingly saline habitats when the EC was lower, but when the EC was higher, the stress of EC may have inhibited most functional traits among the morphological traits. In the Yellow River Delta, the average soil salt content is 0.6% (Fan et al., 2010), close to the EC turning point (≈2.58 ms/cm) of our results. This turning point of soil EC merits further study in the study of the relationship between plant functional traits and environmental factors.

Under high‐salinity environments, plants changed their growth strategy to thicken and strengthen their structures to preserve internal resources in order to survive. The results of the present study verified that the EC of environments was the dominating factor controlling the functional properties of *P. australis* in the Yellow River Delta. While *P. australis* exhibited no obvious specific correlation between SWC and functional traits, except for the leaf water content (Figures 2 and 4), *P. australis* had a wide range of niche adaptations to soil water and had high tolerance to both drought and waterlogged conditions. The results indicated a high adaptability to SWC for the growth of *P. australis*. Soil EC, rather than SWC, was the limiting factor in the Yellow River Delta based on the 96 plots in our research.

### 4.2 The plasticity of traits

Grouping plants by functional trait types rather than by organs could help us more accurately understand the process of plant growth and the response to environmental stress. Functional traits can be divided into morphological traits and physiological traits (Liu & Ma, 2015). Strong plasticity of variability was observed in whole‐plant traits, especially in the morphological traits, and coefficient of variation was the accepted method used to standardize variability among traits with fundamentally different units (Acasuso‐Rivero et al., 2019). Morphological traits involve the shape and structure of each organ, the responses of individuals to environmental changes based on internal genes, and the adaptations of plants to environmental heterogeneity (Hu et al., 2008). In our research, the leaf area, stem internode number, height, and stem basal diameter were attributed to morphological traits. All these traits had high coefficients of variation, which were higher than 20%, while the whole plants had a higher coefficient of variation than 45%, indicating that the morphological growth traits were sensitive to stress due to environmental factors, and *P. australis* was able to adapt to a wide range of niches under salinity stress in the Yellow River Delta.

Physiological traits include enzyme activity, chlorophyll content, and water potential (Gratani, 2014). Some researchers have shown that physiological traits were more susceptible to soil properties with regard to morphological traits (Schneider et al., 2017). Compared with morphological traits, the coefficient of variation values of physiological traits contained the SPAD and leaf water content in our research and were lower than 20%. The lower coefficients of variation indicated relatively lower plasticity and stable intrinsic properties of *P. australis* under various environmental stressors. SLA and leaf thickness, although shown as morphological traits (Smith & Knapp, 2001), were closely related to physiological traits (Wright et al., 2004; Zhang & Luo, 2004) and have obvious influence on SPAD (Marenco et al., 2009). Leaf thickness also plays an important role in leaf and plant functioning and is related to species resource acquisition strategies. Leaf thickness is closely related to other physiological traits such as photosynthetic capacity and respiration (Vile et al., 2005). With the increase in leaf thickness, the mass of per unit area increases, leading to the decrease in SLA. The SLA and leaf thickness remain relatively stable under specific environments and were not only influenced by soil factors, but also restricted by factors including leaf tissue characteristics and chemical composition (Van Arendonk & Poorter, 1994). Lower coefficients of variation in these traits could help *P. australis* resist stress and maintain the stability of plant physiological functions, jointly promoting a wide range of niches under salinity stress in the Yellow River Delta with the higher phenotypic plasticity morphological traits.

### 4.3 The model of *P. australis* life strategies

Three core leaf traits, namely SLA, LDMC, and leaf area, were used as criteria to determine the ecological strategies of individuals (Pierce et al., 2017). Many studies have verified the explanation of CSR strategy classification for community species composition in habitats where species strategy distribution is concentrated (Negreiros et al., 2014, de Paula et al., 2015). CSR strategies are used not only to research the different species in communities but also to investigate the variation of strategies within species under different environmental stresses, especially in widely distributed plants such as *Arabidopsis thaliana* (May et al., 2017; Vasseur et al., 2018).

As another widely distributed species, *P. australis* is distributed in various stages of succession in the Yellow River Delta. The CSR strategies can be used to explore the dynamic processes of *P. australis* under different environment stresses. In our research, variation in ecological strategies along the S–C axis was found in *P. australis*. The plants with strong salinity stress tolerance under the S/CS strategy have more advantages, and the plants near the S strategy end have greater survival opportunities. S is the dominant strategy for *P. australis* in the Yellow River Delta. Plants near the S strategy end have lower LDMC and SLA, while plants near the C strategy end have higher SLA traits, representing resource acquisition capacity, than those near the S strategy end. We believe that environmental factors partly determined the C or S strategy of *P. australis*, while with the decrease in environmental stress, the main strategy gradually shifted from S to C. Under the condition of high electrical conductivity, *P. australis* improved its water acquisition ability by increasing indicators such as LWC and leaf thickness (Table 1; Figure 3). With the increase in plant tolerance to stress, more resources are used to resist external stress, and the survival strategy is inclined toward the S strategy. For the C strategy, plant selection and growth are mainly aimed at rapidly occupying resources, and a relatively large SLA is needed to grow rapidly (Xu et al., 2019). With the decrease in soil salinity and the increase in SWC, the strategy of *P. australis* in the Yellow River Delta was accompanied by increased SLA and leaf area (Table 1; Figure 3) for its growth in order to obtain resources rapidly, while its survival strategy gradually moved to the C strategy (Figure 4). This proved that the functional traits of *P. australis* were more sensitive to salt content. The variability of strategies for *P. australis* indicated that in the high‐salinity area, the competition (C) and stress tolerator (S) strategies were the main strategies that affected the growth and distribution of *P. australis*. With the decrease in environmental stress, the main strategy gradually moved from S to C.

In the Yellow River Delta, *P. australis* plants have a minor R strategy, but under lower environmental stress, the proportion of R gradually increases (Figure 4). Ruderal (R) plants are typically associated with a short life cycle, low LDMC, and high SLA and presumably have a high metabolic rate and low tissue protection (Grime, 1977). Plants under the R strategy are adapted to environments with strong disturbance and tend to invest many resources in reproduction to offset the effects of disturbances on the population, thereby achieving the purpose of survival and continuation of the population (Pierce et al., 2017). As a perennial herb, *P. australis* tends to grow rapidly and spread through underground stems, forming a single community, which is common in the Yellow River Delta. Due to the habits of *P. australis*, the R strategy rarely appears in the Yellow River Delta. However, the increased proportion of R is worth attention in future studies, with a possible focus on whether the *P. australis* will lose its dominant species status with the R strategy in the Yellow River Delta.

## CONFLICT OF INTEREST

The authors declare no conflict of interest in the publication of this paper.

## AUTHOR CONTRIBUTIONS

**Dayou Zhou:** Conceptualization (equal); Data curation (equal); Formal analysis (equal); Investigation (equal); Methodology (equal); Writing‐original draft (equal); Writing‐review & editing (equal). **Yuehan Ni:** Conceptualization (equal); Data curation (equal); Formal analysis (equal); Methodology (equal); Writing‐original draft (equal). **Xiaona Yu:** Conceptualization (equal); Data curation (equal); Formal analysis (equal); Methodology (equal); Validation (equal); Writing‐review & editing (lead). **Kuixuan Lin:** Funding acquisition (equal); Investigation (equal); Project administration (equal); Writing‐review & editing (equal). **Ning Du:** Formal analysis (equal); Investigation (equal); Methodology (equal); Writing‐review & editing (equal). **Lele Liu:** Formal analysis (equal); Methodology (equal); Visualization (equal); Writing‐review & editing (equal). **Xiao Guo:** Formal analysis (equal); Methodology (equal); Writing‐review & editing (equal). **Weihua Guo:** Conceptualization (equal); Data curation (equal); Funding acquisition (lead); Methodology (equal); Project administration (lead); Writing‐original draft (supporting); Writing‐review & editing (equal).

## Supporting information

App S1

## Data Availability

Plant functional traits and environmental factors data input files are available at: https://doi.org/10.5061/dryad.x3ffbg7j4.
